# Public Health Data Exchange Through Health Information Exchange Organizations: National Survey Study

**DOI:** 10.2196/64969

**Published:** 2024-11-15

**Authors:** Sarah Rosenthal, Julia Adler-Milstein, Vaishali Patel

**Affiliations:** 1 Division of Clinical Informatics and Digital Transformation, University of California, San Francisco, San Francisco, CA, United States; 2 Technical Strategy and Analysis Division, Office of the National Coordinator for Health Information Technology, Washington, DC, United States

**Keywords:** public health informatics, health information exchange, health information technology, data exchange, health information, national survey, surveillance, United States, PHA, HIO, public health agency, health information exchange organization

## Abstract

**Background:**

The COVID-19 pandemic revealed major gaps in public health agencies’ (PHAs’) data and reporting infrastructure, which limited the ability of public health officials to conduct disease surveillance, particularly among racial or ethnic minorities disproportionally affected by the pandemic. Leveraging existing health information exchange organizations (HIOs) is one possible mechanism to close these technical gaps, as HIOs facilitate health information sharing across organizational boundaries.

**Objective:**

The aim of the study is to survey all HIOs that are currently operational in the United States to assess HIO connectivity with PHAs and HIOs’ capabilities to support public health data exchange.

**Methods:**

Drawing on multiple sources, we identified all potential local, regional, and state HIOs that were operational in the United States as of March 1, 2022. We defined operational as HIOs that facilitated exchange between at least 2 independent entities. We fielded a survey among our census list of 135 HIOs in January-July 2023. The survey confirmed HIO status as well as captured organizational demographics and current and potential support for PHAs. We report descriptive statistics on HIO demographics and connectivity with PHAs. We also include results on services and data available to support PHAs, funding sources to support public health reporting, and barriers to public health reporting. Of the 135 potential HIOs that received the survey, 90 met our definition of an HIO, and 77 completed the survey, yielding an 86% response rate.

**Results:**

We found that 66 (86%) of HIOs in 45 states were electronically connected to at least 1 PHA, yielding 187 HIO-PHA connections across all HIOs. Among HIOs connected to PHAs, the most common type of public health reporting supported by HIOs was immunization registry (n=39, 64%), electronic laboratory result (n=37, 63%), and syndromic surveillance (n=34, 61%). In total, 58% (n=38) of HIOs connected to PHAs provided data to address COVID-19 information gaps, and an additional 30% (n=20) could do so. The most common types of data provided to PHAs were hospitalization information (n=54, 93%), other demographic data (n=53, 91%), health information (eg, chronic health conditions; n=51, 88%), and hospital laboratory results (n=51, 88%). A total of 64% (n=42) of HIOs provided at least 1 type of data analytic service to PHAs to support COVID-19 pandemic response efforts. Top HIO reported barriers to support PHA activities included limited PHA funding (n=21, 32%) and PHAs’ competing priorities (n=15, 23%).

**Conclusions:**

Our results show that many HIOs are already connected to PHAs and that they are assuming an emerging role to facilitate public health reporting. HIOs are well-positioned to provide value-added support for public health data exchange and address PHAs’ information gaps, as ongoing federal efforts to modernize public health data infrastructure and interoperability continue.

## Introduction

The ability to collect and analyze timely and complete data is essential to public health efforts to control disease outbreaks. The COVID-19 pandemic revealed major gaps in public health agencies’ (PHAs) data and reporting infrastructure. These included outdated information systems, insufficient technical expertise (particularly due to staff turnover), and lack of reporting standards and submission templates [1-3]. Such shortcomings limited the ability of public health officials to conduct disease surveillance, particularly among racial or ethnic minorities disproportionally affected by the pandemic [4]. In response, the Centers for Disease Control and Prevention (CDC) expanded the Data Modernization Initiative (DMI), a multiyear, multibillion-dollar effort to increase PHAs’ access to better, more timely, and actionable data to improve health equity and protect public health [5].

A key priority of DMI is to “accelerate data into action,” which involves addressing gaps in public health data, reducing the complexity of exchange, and providing more actionable, timely data [5,6]. One mechanism to accomplish this could be leveraging existing health information exchange organizations (HIOs). The passage of the Health Information Technology for Economic and Clinical Health Act in 2009 established broad-based electronic health information exchange as a national priority. However, given the complex ecosystem of health care delivery organizations and electronic health record vendors, HIOs emerged to facilitate health information sharing across organizational boundaries by creating interfaces through which providers can exchange data with other participating providers and broader stakeholders such as payers, laboratories, and PHAs. Through their exchange network, HIOs may also build large master patient indexes (MPIs) to serve as repositories of data, including data that are relevant to public health reporting, and could help address gaps in health equity–related information. Furthermore, HIOs could be strong partners for PHAs because they typically operate as state, local, or regional entities with a deep understanding of, and experience with, local health care environments. HIOs may be well-positioned to actively support electronic reporting to PHAs, improve data quality, and produce unique insights on a community’s longitudinal health and specific trends, particularly within underresourced populations through combining clinical and demographic data.

Federal policy has sought to decrease PHAs’ burden related to data exchange by enabling connections with an array of providers through health information networks rather than relying on one-to-one connections with each individual provider. During the COVID-19 pandemic, the Office of the National Coordinator for Health Information Technology’s (ONC) Strengthening the Technical Advancement and Readiness of Public Health via Health Information Exchange Program (STAR HIE Program) enabled HIOs to develop services to support PHAs’ need for timely and high-quality information [7]. Relatedly, the COVID-19 Immunization Data Exchange, Advancement, and Sharing Program (IDEAS Program) provided financial and technical assistance to connect HIOs and state health agency immunization information systems [8,9]. Looking beyond the pandemic, efforts are now focused on the participation of health information networks in the Trusted Exchange Framework and Common Agreement (TEFCA). TEFCA is a national framework that has developed baseline governance, legal, and technical requirements to enable secure information sharing across different networks nationwide [10]. Public health is among the 6 exchange purposes currently authorized under TEFCA, and there are plans to support specific types of public health reporting, such as electronic case reporting, and to facilitate exchange between PHAs, directly supporting CDC’s DMI efforts [11].

To generate an understanding of the current state and future potential of HIOs to support PHA needs, a national survey of HIOs was conducted to assess (1) current connectivity between HIOs and PHAs, including participation in TEFCA; (2) services provided to facilitate public health reporting; (3) services provided to support PHAs’ COVID-19 response; (4) how HIOs address information gaps for PHAs; (5) HIO funding sources to support public health services; and (6) HIO-reported barriers to supporting PHAs. Our results serve to inform how the CDC’s DMI and TEFCA can better engage HIOs to achieve their objectives.

## Methods

### Identifying HIOs

We sought to survey all local, regional, and state HIOs operating in the United States and its territories that supported live electronic health information exchange across their network as of March 1, 2022, and that facilitated exchange between independent entities (defined as institutions with no financial relationship with exchange occurring between entities where at least one is independent of the others). We excluded those that we determined via a web search to be no longer operational or to have merged with another HIO. To build our census list, we relied on contacts compiled from previous national surveys conducted by our research team biennially between 2007 and 2019. We then worked with Civitas Networks for Health (Civitas), which is a national HIO member organization with more than 50 HIOs, to update the distribution list. Our final list consisted of 135 potential HIOs. To be consistent with previous years, we did not include HIO networks led by single vendors or a consortium of vendors, such as Epic’s Care Everywhere Network or the CommonWell Health Alliance.

### Survey Instrument Development

We started with the survey instrument fielded in 2019 and added a new section that captured public health reporting capabilities. The updated instrument consisted of screening questions to determine eligibility to participate in the survey followed by 5 sections: organizational demographics, public health reporting, implementation and use of standards, network-to-network connectivity and TEFCA, and information blocking. Screening questions asked respondents to determine whether, as of March 1, 2022, the organization was supporting operational electronic health information exchange among independent entities. Respondents whose organizations met these criteria were prompted to complete the rest of the survey. The organizational demographics section captured general HIO characteristics such as the types of services provided, governance details, number of unique individuals within their MPI, and geographic coverage. For geographic coverage, we first asked in which states the HIO operated. For each state that they indicated, we asked which health service areas the HIO covered. In the network-to-network connectivity and TEFCA section, we asked about plans to participate in TEFCA—planning, unsure, or not planning—and their participants’ abilities to fulfill exchange purposes included in TEFCA (government benefits determination, public health, payment, treatment, health care operations, and individual access).

The new public health section first asked if the HIO was currently connected to any PHA. We defined connectivity as a PHA providing data to or receiving data from the HIO. Those not connected to any PHA were screened out of the section. If they were connected to a PHA, they were asked to list the specific PHAs that they were connected to (with a maximum of 5 entities that could be listed), the type of PHA (state, local or county, or other), and the nature of the connections (provides data, receives data, or bidirectional). We then asked HIOs to report the status (in production, in testing or planning, available but PHA not able or willing, or not available) of services to providers to facilitate reporting to PHAs and to report services provided to PHAs to support COVID-19 response efforts (7 total—eg, dashboarding, outbreak monitoring, and alerting). We also asked what types of data HIOs provided to supplement data reported to PHAs (8 total—eg, hospital laboratory results and race or ethnicity), funding sources for public health data exchange (7 total—eg, participant fees and federal funding), and barriers to supporting public health reporting (11 total—eg, PHA lacks staffing and limited funding from HIO participants). For each of the 11 barriers listed, HIOs were asked to report the extent to which they experienced it—ranging from “not at all” to “to a great extent.” (For this paper, we did not use questions in the remaining 2 sections—implementation and use of standards and information blocking).

We completed 2 rounds of pilot-testing (first with 4 HIOs and then with 3 HIOs), refining the survey based on their input after each round. A Microsoft Word version of the final survey instrument is available in Multimedia Appendix 1.

### Survey Administration

We fielded the closed survey via the Qualtrics XM web-based platform (Qualtrics International) between January and July 2023, emailing the survey to the director of each HIO, the respondent from previous HIO surveys, or the person in the organization indicated by the contact confirmation form (fielded 1 month before the survey asking the respondent from the 2019 survey if they were still the correct respondent for the survey and if not, to please list an alternate). Qualtrics XM automatically generated personalized, single-use links for each HIO in our sample, ensuring that 1 respondent could not submit multiple entries.

We initially recruited HIOs to complete the survey via emails sent through Qualtrics XM. After 2 rounds of follow-up emails sent in Qualtrics XM, we followed up with emails from the principal investigator (JA-M) and from the CEO of Civitas. Additionally, we called nonrespondents to ensure they had received the survey and to answer any questions. Nonrespondents received 5 follow-up email messages and 1 phone call.

When taking the survey, respondents were able to review and change their answers through a back button. Due to the branching logic of the survey, items could not be randomized or alternated. Branching logic was used to ensure that respondents viewed only those items relevant to their operations.

Of the 135 organizations that received the survey, 45 were determined to be ineligible as they no longer were operational, did not pursue live health information exchange, did not facilitate exchange between independent entities, or had merged with another HIO. Of the 90 remaining HIOs that met our inclusion criteria, 77 completed or had sufficient partial completion (defined as more than 50%) of the survey, resulting in an 86% response rate. This response rate is consistent with prior HIO surveys [12-17].

### Analysis

Data were cleaned and analyzed in Stata (version 18.0; StataCorp). We conducted descriptive analyses limited to the HIOs that reported being connected to at least 1 PHA. First, we produced descriptive statistics on the number of unique individuals HIOs reported in their MPI as well as the total (not deduplicated) number of individuals across all HIOs. Second, to assess the connectivity between HIOs and PHAs, we counted the total number of connections they reported (within the maximum of 5 PHA connections). The type of connection was examined both at the HIO level (eg, the percent of HIOs that had a bidirectional connection with a PHA) and the HIO-PHA dyad level (eg, the percent of total connections that were bidirectional). Third, to assess geographic coverage, we combined HIO-PHA connection dyads with data on the states HIOs reported operating in. For HIOs that operated in multiple states, the correct state was manually coded based on the name of the PHA listed. We then created a map depicting the density of these connections using counts of HIO-PHA connections aggregated by state.

Finally, we produced descriptive statistics for responses to questions on (1) services to facilitate public health reporting to PHAs; (2) services provided to PHAs to support COVID-19 response efforts; (3) availability of data to address pandemic-related and health equity–related information gaps; (4) plans to participate in TEFCA and the percent of HIOs whose participants were currently able to make or respond to requests for information related to public health, as defined by TEFCA; (5) funding sources to support public health reporting; and (6) barriers to public health reporting. To describe barriers to public health reporting, we reported on the percentage of HIOs that experienced the barrier “to a great extent.”

### Ethical Considerations

This study received exempt designation from the University of California, San Francisco institutional review board (IRB #20‐32169). Participants were notified in recruitment language and at the start of the web-based survey that participation was voluntary, and they were informed of the length of the survey, data access policies, the purpose of the study, and the contact information for the investigator and institutional review board. Individual responses were stored securely according to institutional policy. Study data are presented only in the aggregate, and no individual participants or their organizations are named. Participants were offered a financial incentive (a US $50 gift card if they completed the entire survey and a US $10 gift card if they answered the screening questions but screened out).

## Results

### Characteristics of HIOs Electronically Connected to PHAs

We found that 66 (86%) HIOs were electronically connected to a PHA (ie, at least 1 PHA provides data to or receives data from the HIO). On average, HIOs connected to PHAs reported that they had over 9 million individuals within their MPI, which assigns patients a unique identifier to enable electronic exchange and is an indicator of HIO size (Table 1). Together, HIOs that were connected to PHAs had a total of 539 million individuals in their MPIs, reflecting overlap in individuals across MPIs.

**Table 1.** Health information exchange organization (HIO) connections with public health agencies (PHAs) and broader networks. Results from a national survey of HIOs fielded January-July 2023 (n=66 HIOs).

Demographics for HIOs connected to PHAs	Values
**Public health connections for HIOs with at least 1 connection (maximum: 5 connections)**	
Mean (SD)	3.06 (1.64)
Median (IQR)	3.00 (1-5)
**Individuals in master patient index for HIOs with at least 1 connection**	
Mean (SD)	9,628,424 (11,514,237)
Median (IQR)	5,500,000 (3,000,000-12,000,000)
**State versus local or county PHA connections (n=66 HIOs connected to at least 1 PHA), n (%)**	
Only state	32 (48)
Only local	7 (11)
Both state and local	27 (41)
**HIO-PHA exchange connection at the HIO level (n=66 HIOs connected to at least 1 PHA), n (%)**	
Bidirectional exchange with at least 1 PHA	52 (79)
HIO receives data from PHA only	4 (6)
HIO sends data to PHA only	10 (15)
**HIO-PHA exchange connection at the dyad level (n=187 total connections between HIOs and PHAs), n (%)**	
Bidirectional exchange	85 (45)
HIO receives data from PHA only	30 (16)
HIO reports data to PHA only	72 (39)
**TEFCA^a^ network participation (n=66 HIOs connected to at least 1 PHA), n (%)**	
Plan to participate in TEFCA	41 (62)
Plan to participate in TEFCA and have the capability to address the TEFCA public health use case	34 (52)

a TEFCA: Trusted Exchange Framework and Common Agreement.

### Connectivity Between HIOs and PHAs

Each HIO had on average 3 connections to state or local PHAs, yielding a total of 187 HIO-PHA connections across all HIOs. Almost half (n=32, 48%) of HIOs were connected only to state PHAs, with 41% (n=27) connected to both state and local PHAs and 11% (n=7) connected only to local PHAs. Most HIOs (n=52, 79%) had bidirectional exchange in place with at least 1 PHA (Table 1). When examining this based on the 187 HIO-PHA connections, 45% (n=85) involved bidirectional exchange.

**Figure 1.**
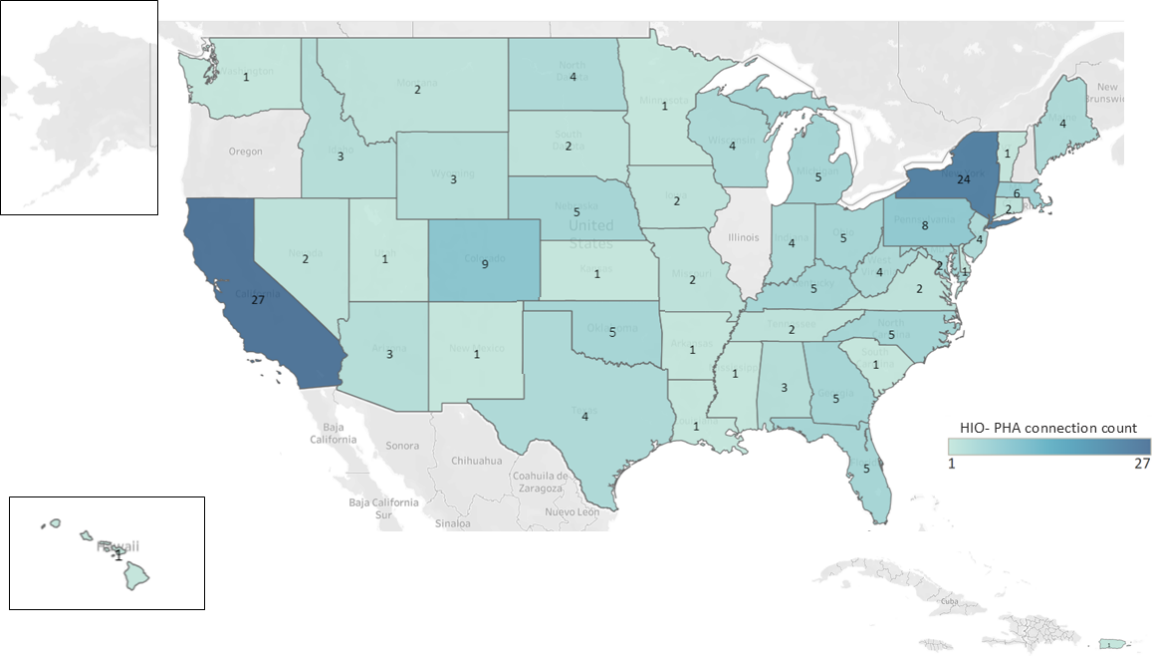
Map of HIO-PHA connection density by state. Results from a national survey of HIOs fielded January-July 2023. (n=187 connections and n=12 “other” connections, eg, federal, registry, and unspecified). Certain states (such as New York and California) with high numbers of operating HIOs may have more connections. HIO: health information exchange organization; PHA: public health agency.

In total, 45 states as well as Puerto Rico and Washington DC had at least 1 connection between an HIO and a PHA (Figure 1). California and New York had the most HIO-PHA connectivity, with the largest number of operating HIOs (11 and 7, respectively) and 27 and 24 HIO-PHA connections, respectively.

### Services Provided by HIOs for Public Health Reporting and the COVID-19 Response

**Figure 2.**
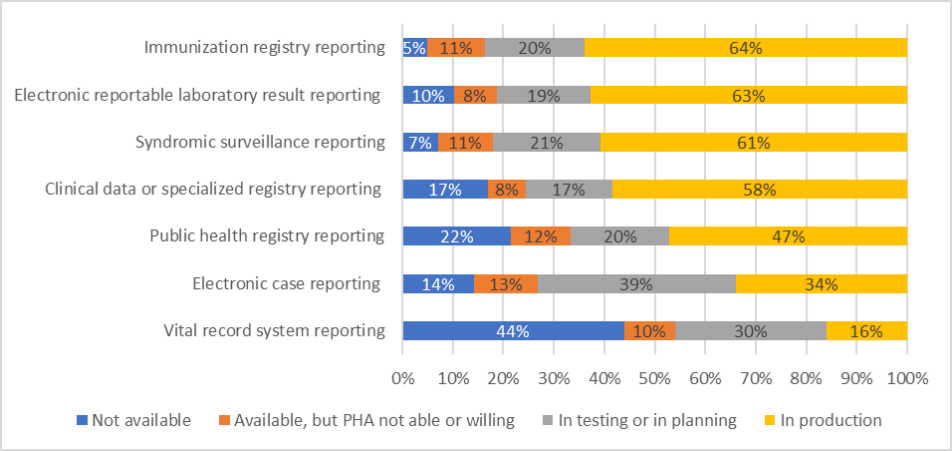
Services offered to participating health care providers to support public health reporting. Results from a national survey of HIOs fielded January-July 2023 (n=66 HIOs). HIO: health information exchange organization; PHA: public health agency.

HIOs’ provision of services to enable their participating providers to electronically report public health data to PHAs varied by type of reporting (Figure 2). The most common types of public health reporting currently in production by HIOs were immunization registry reporting (n=39, 64%), electronic laboratory result reporting (n=37, 63%), and syndromic surveillance reporting (n=34, 61%). An additional 19%‐21% (n=11-12) of HIOs were in the planning or testing phases for these public health reporting activities. Although fewer HIOs were currently in production with electronic case reporting (n=19, 34%) or vital record system reporting (n=8, 16%), additional HIOs reported being in the testing or planning stages with these types of reporting.

In addition to supporting public health reporting between health care providers and PHAs, HIOs provided additional services to PHAs to support the COVID-19 response. Almost one-third (n=21, 32%) of HIOs connected to PHAs provided dashboards or other visualization services, 30% (n=20) used their MPI to support deduplication of public health data, and 26% (n=17) provided analytic and data quality support to PHAs (Table 2). The least commonly provided services to PHAs related to outbreak monitoring and alerting (n=11, 17%) and public health policy impact monitoring (n=5, 8%). Overall, about two-thirds of HIOs (n=42, 64%) connected to PHAs provided at least 1 of these services to PHAs.

**Table 2.** Services provided to public health agencies (PHAs) to support the COVID-19 response. Results from a national survey of health information exchange organizations (HIOs) fielded January-July 2023 (n=66 HIOs).

Services provided to PHAs to support COVID-19 response	Values, n (%)
Any of the below	42 (64)
Dashboarding and data visualization assistance	21 (32)
Use of HIO master patient indexes to support public health deduplication or other services	20 (30)
Analytic and data quality support	17 (26)
Process automation	15 (23)
Outbreak monitoring and alerting	11 (17)
Public health policy impact monitoring	5 (8)

### Addressing Gaps in Information

Most HIOs indicated that they currently provided data to address PHA gaps in missing information related to COVID-19 reporting (n=38, 58%) or could do so (n=20, 30%; Table 3). Among those HIOs that currently provided data to PHAs to address gaps in information, a majority (n=31, 81%) reported that PHAs often electronically received or queried these types of data. Of those HIOs that did or could provide data, available data elements included hospitalization information (n=54, 93%), demographic information other than race or ethnicity (n=53, 91%), health information (n=51, 88%), hospital laboratory results (n=51, 88%), race or ethnicity data (n=50, 86%), and updated contact information (n=50, 86%).

**Table 3.** Provision of data by health information exchange organizations (HIOs) to public health agencies (PHAs) to address pandemic-related and health equity–related information gaps. Results from a national survey of HIOs fielded January-July 2023 (n=66 HIOs).

	Values, n (%)
**Do you currently provide data to PHAs to fill gaps in COVID-19–related data (eg, missing demographic information)?**	
Yes	38 (58)
No but could do so	20 (30)
No and could not do so	1 (2)
**If yes or could do so (n=58), what types of data are or could be provided?**	
Hospitalization information	54 (93)
Other demographics	53 (91)
Health information (eg, chronic health conditions)	51 (88)
Hospital laboratory results	51 (88)
Race or ethnicity	50 (86)
Updated contact information	50 (86)
Commercial laboratory results	46 (79)
Immunization data	41 (71)
Other	4 (7)
**Among HIOs providing data to PHAs to fill gaps in COVID-19–related data (n=38), what is the frequency of PHAs electronically receiving or querying these types of data?**	
Often	31 (81)
Sometimes	6 (16)
Rarely	1 (3)
Never	0 (0)

### TEFCA Participation

Almost two-thirds of HIOs that were connected to PHAs (n=41, 62%) planned to participate in TEFCA, and over half (n=34, 52%) of both planned to participate and had the capability for the TEFCA public health exchange purpose, as measured by their participants’ ability to make or respond to public health–related requests for information (Table 1).

### Funding Sources

HIOs reported 3 main funding sources to support their services to PHAs: participant fees, state, and federal grants. While HIOs mostly cited participant fees (which could include PHAs if they participated in the HIO) as a funding source (n=30, 45%), state funding represented another major source, with 36% (n=24) of HIOs using state Medicaid funding, 35% (n=23) using other state funding, and 30% (n=20) reporting fees paid by the state to support the services they provided to PHAs (Table 4). Federal sources of funding included the STAR HIE Program (n=14, 21%), Coronavirus Aid, Relief, and Economic Security Act funding (n=7, 11%), and other federal sources (n=12, 18%).

**Table 4.** Health information exchange organizations’ (HIOs’) funding sources for public health reporting. Results from a national survey of HIOs fielded January-July 2023 (n=66 HIOs).

HIOs’ funding sources to support public health reporting	Values, n (%)
Fees paid by participants	30 (45)
State Medicaid funding	24 (36)
Other state funding, including from state health department	23 (35)
Fees paid by state health department	20 (30)
STAR HIE^a^ program	14 (21)
Other federal funding	12 (18)
Do not receive any funding specifically to support public health reporting	9 (14)
CARES^b^ Act funding	7 (11)
Other	6 (9)

a STAR HIE Program: Strengthening the Technical Advancement and Readiness of Public Health via Health Information Exchange Program.

b CARES: Coronavirus Aid, Relief, and Economic Security.

### Barriers to Supporting PHAs

In total, 32% (n=21) of HIOs cited limited funding from PHAs as a barrier to public health reporting “to a great extent,” and 14% (n=9) cited limited funding from their participants (Table 5). HIOs also indicated PHAs’ other priorities (n=15, 23%) and lack of staffing (n=11, 17%) as barriers. This was followed by PHAs lacking the technical capability to process (n=10, 15%) and receive (n=7, 11%) messages from the HIOs.

**Table 5.** Health information exchange organization (HIO) reported barriers to public health reporting. Results from a national survey of HIOs fielded January-July 2023 (n=66 HIOs)^a^.

Barriers to public health reporting	Values, n (%)
Limited funding from PHA^b^	21 (32)
PHA has other priorities	15 (23)
PHA lacks staffing	11 (17)
PHA lacks technical capability to process messages from your HIO	10 (15)
Limited funding from your HIO participants	9 (14)
Patient consent model hinders data exchange with PHA	9 (14)
State statutes or regulations limit PHA participation with HIO	7 (11)
PHA lacks technical capability to receive messages from your HIO	7 (11)
Need for data use agreements for public health data	6 (9)
Other technical limitations on part of PHA	4 (6)
Low return on investment to your HIO	3 (5)

a Percentage of HIOs that reported experiencing the barrier to “a great extent.”

b PHA: public health agency.

## Discussion

### Principal Findings

Our study captures timely national data on HIO support for PHAs. Based on our results, HIOs appear to be providing value-added services that could be expanded to further support public health data exchange needs, as PHAs modernize their infrastructure. We found that a majority of HIOs were connected to at least 1 state or local PHA across most states within the United States. Among HIOs connected to PHAs, about half enabled or planned to enable public health reporting between health care providers and PHAs, and over half provided services to PHAs that addressed gaps in information and supported data analytics. However, barriers to further expanding their support likely need to be addressed to surpass the current level of engagement.

We found that the type and nature of the HIO-PHA connections varied. HIOs reported fewer connections to local PHAs, and only half reported bidirectional exchanges across their broader set of PHA connections. These findings suggest that there is a need to expand the degree of connectivity across existing connections to increase bidirectional exchange and expand local PHA connections. Plans to participate in TEFCA among those connected to PHAs were high, suggesting that these HIOs could help support public health data exchange through this mechanism as TEFCA expands.

A primary, though still nascent, role that HIOs connected to PHAs serve is enabling public health reporting between participating health care providers and PHAs, particularly related to reportable laboratory results, syndromic surveillance, and immunization reporting. The provision of these services will likely expand given that some HIOs reported these types of reporting are in testing or planning phases, and only few reported inability or lack of willingness on the part of PHAs to engage with them. The ONC IDEAS Program, which specifically sought to establish and scale the sharing of vaccine data between state immunization information systems and HIOs, may have helped HIOs to support immunization exchange, which was the most frequent type of public health reporting.

Given HIOs’ access to millions of records in their MPIs and shared data from their participants, leveraging these data for a variety of public health purposes is promising. About two-thirds of HIOs connected to PHAs supported at least 1 type of data analytic service, such as dashboards, and data processing, such as deduplication through the use of their MPIs. For example, a Maryland HIO linked COVID-19 electronic laboratory reports to their MPI to support outbreak investigations [18]. In Indiana, the statewide HIO supported public health surveillance and response by sharing timely, accurate data via a population-level dashboard [19].

HIOs’ MPIs and shared data from participants were also leveraged to address data gaps. Most HIOs connected to PHAs reported that they currently or could provide data to fill gaps related to key clinical information such as hospitalization data, along with demographic information, including race or ethnicity and contact information. These latter data were key pieces of information that PHAs were often missing, impeding their response to the pandemic, particularly for marginalized populations, and may help the CDC’s DMI effort to improve the collection of equity-related data [20].

While HIOs’ current capabilities to support PHAs are encouraging, they indicated several barriers to doing so. HIOs reported that the primary funding for public health reporting came from participant fees and state funding. Yet, one-third of HIOs cited limited funding from PHAs and HIO participants as major barriers to enabling public health reporting. Although about one-fifth of HIOs relied on the STAR HIE Program and, to a lesser extent, the Coronavirus Aid, Relief, and Economic Security Act to support public health reporting activities, these federal funding sources are no longer available. Findings from an evaluation of the original STAR HIE Program grantees showed that funding not only helped HIOs build capabilities to support public health use cases but also incentivized partnerships between HIOs and PHAs that would extend to future collaborations [21]. In its absence, funding from CDC’s DMI program may help sustain and expand HIOs’ support for public health reporting and other services.

Other barriers cited by HIOs relate to PHA capacity to engage in electronic public health reporting, including a limited workforce, competing priorities, and technical capabilities to process and receive messages from HIOs. To address staffing issues, both CDC and ONC have programs that seek to bolster the public health workforce [22,23]. The ONC program has provided US $75 million to educational institutions seeking to increase the number of public health professionals trained in public health and informatics, particularly among underresourced communities. While PHAs understandably dealt with many competing priorities during the pandemic along with outdated IT systems, CDC’s DMI and other public health infrastructure grants are providing substantial resources to PHAs to advance their health IT infrastructure to enable data exchange and analytics [23-25]. Recently proposed regulations call for updating standards used to exchange public health data in both electronic health record systems and public health data systems, which if implemented would bolster the capabilities of these systems to support the seamless exchange of public health data through HIOs and other networks [26,27]. Only a minority of HIOs cited barriers related to patient consent models, and state laws or regulations hindering connections between HIOs and PHAs. However, inconsistencies in consent requirements between what can be shared with a PHA without patient consent versus what can be shared with a HIO, particularly in states requiring opt-in consent for HIOs, can be complex [9]. As these federal initiatives are implemented, it will be important to track to what degree they address the identified barriers and increase HIO-PHA engagement on public health reporting and analytics.

### Limitations

Our study has several limitations. HIO survey data are self-reported and are not independently verified. Reporting bias may overestimate the degree to which support for public health reporting and other data exchange is occurring. However, due to our 86% response rate and the restricted number of PHAs to which HIOs could report being connected, we likely underestimate the number of individual HIO-PHA connections. Additionally, our findings are from the HIO perspective and do not capture the perspective of their connecting PHA partners. PHA perspectives on the barriers, connectivity, and services provided by HIOs would be valuable to gain a complete understanding of the role of HIOs in enabling public health data exchange. Our analysis was limited to descriptive statistics and does not include any inferential statistics. As such, results may not be generalizable beyond the set of HIOs that responded to our survey.

### Conclusions

Our findings reveal that many HIOs already have connections with PHAs and that they are assuming an emerging role to both facilitate data exchange between health care providers and PHAs and to help PHAs directly by addressing gaps in information and providing analytic services. Many HIOs that are connected to PHAs also plan to participate in TEFCA and could further enable public health data exchange through that mechanism. Modernization of the public health data systems and the informatics training of the public health workforce may reduce some key barriers to fostering greater partnership between PHAs and HIOs. Concerns regarding HIOs’ ability to sustain and further expand their efforts to facilitate PHA connectivity will need to be addressed, as efforts are made to further advance public health data exchange nationwide.

## Supplementary material

10.2196/64969 Multimedia Appendix 1 2023 Health information exchange organization survey instrument.

## Abbreviations

CDC: Centers for Disease Control and Prevention
DMI: Data Modernization Initiative
HIO: health information exchange organization
IDEAS Program: COVID-19 Immunization Data Exchange, Advancement, and Sharing Program
MPI: master patient index
ONC: Office of the National Coordinator for Health Information Technology
PHA: public health agency
STAR HIE Program: Strengthening the Technical Advancement and Readiness of Public Health via Health Information Exchange Program
TEFCA: Trusted Exchange Framework and Common Agreement

## References

[1] Basit MA, Lehmann CU, Medford RJ (2021). Managing pandemics with health informatics: successes and challenges. Yearb Med Inform.

[2] Jiang JX, Cram P, Qi K, Bai G (2024). Challenges and dynamics of public health reporting and data exchange during COVID-19: insights from US hospitals. Health Aff Sch.

[3] Baker M, Ivory D (2021). Why public health faces a crisis across the U.S. The New York Times.

[4] Huyser KR, Horse AJY, Kuhlemeier AA, Huyser MR (2021). COVID-19 pandemic and Indigenous representation in public health data. Am J Public Health.

[5] (2024). Data Modernization Initiative. Centers for Disease Control and Prevention.

[6] (2024). Overview: the public health data strategy. Centers for Disease Control and Prevention.

[7] STAR HIE Program. HealthIT.gov.

[8] ONC Tech Forum: modernizing public health data exchange: lessons learned and tools for the road ahead. HealthIT.gov.

[9] (2023). Immunization information systems and health information exchanges. Association of State and Territorial Health Officials.

[10] (2023). Trusted Exchange Framework and Common Agreement (TEFCA). Office of the National Coordinator for Health Information Technology.

[11] Layden JE, Swain MJ, Brennan N, Tripathi M (2024). Plugging public health data into the health IT ecosystem to protect national health. NEJM Catalyst.

[12] Adler-Milstein J, McAfee AP, Bates DW, Jha AK (2007). The state of regional health information organizations: current activities and financing. Health Aff (Millwood).

[13] Adler-Milstein J, Bates DW, Jha AK (2009). U.S. regional health information organizations: progress and challenges. Health Aff (Millwood).

[14] Adler-Milstein J, Bates DW, Jha AK (2011). A survey of health information exchange organizations in the United States: implications for meaningful use. Ann Intern Med.

[15] Adler-Milstein J, Bates DW, Jha AK (2013). Operational health information exchanges show substantial growth, but long-term funding remains a concern. Health Aff (Millwood).

[16] Adler-Milstein J, Lin SC, Jha AK (2016). The number of health information exchange efforts is declining, leaving the viability of broad clinical data exchange uncertain. Health Aff (Millwood).

[17] Adler-Milstein J, Garg A, Zhao W, Patel V (2021). A survey of health information exchange organizations in advance of a nationwide connectivity framework. Health Aff (Millwood).

[18] Feldman KA, Hanks A, Williams TW (2023). A state health department and health information exchange partnership: an effective collaboration for a data-driven response for COVID-19 contact tracing in Maryland. Sex Transm Dis.

[19] Dixon BE, Grannis SJ, McAndrews C (2021). Leveraging data visualization and a statewide health information exchange to support COVID-19 surveillance and response: application of public health informatics. J Am Med Inform Assoc.

[20] (2023). DMI and health equity. Centers for Disease Control and Prevention.

[21] Adler-Milstein JR (2023). Program evaluation for the Strengthening the Technical Advancement and Readiness of Public Health via Health Information Exchange (STAR HIE)—cooperative agreement program. Office of the National Coordinator for Health Information Technology.

[22] Public Health Informatics & Technology (PHIT) Workforce Development Program. HealthIT.gov.

[23] (2024). Public Health Infrastructure Grant. Centers for Disease Control and Prevention.

[24] Public Health Infrastructure Grant. Public Health Data Modernization Implementation Centers.

[25] (2023). Building the right foundation. Centers for Disease Control and Prevention.

[26] Health data, technology, and interoperability: patient engagement, information sharing, and public health interoperability. Office of the National Coordinator for Health Information Technology.

[27] (2024). Health Data, Technology, and Interoperability (HTI-2): patient engagement, information sharing, and public health interoperability proposed rule. Office of the National Coordinator for Health Information Technology.

