# Supplementary material for: Public Health Data Exchange Through Health Information Exchange Organizations: National Survey Study
Source: JMIR Public Health Surveill. 2024 Nov 15;10:e64969. doi: 10.2196/64969 (PMC11611779; doi:10.2196/64969)
Supplement: Multimedia Appendix 1 [file publichealth-v10-e64969-s001.docx]

Form Approved
OMB No. 0955-0019
Exp. Date 11/30/2025

**2023 Health Information Organization (HIO) Survey and Civitas Member Survey**

 The nationwide survey of HIOs is being led by Civitas Networks for Health in collaboration with the University of California, San Francisco and is sponsored by the Office of the National Coordinator for Health IT (ONC).  **Participation is completely voluntary and will contribute to a research study.**Thank you in advance for your time.
  
The survey includes questions in five broad areas:

1. Organizational Demographics
2. Public Health Reporting
3. Implementation/Use of Standards
4. Network-to-Network Connectivity and TEFCA
5. Information Blocking

There is a sixth section of questions, only asked of Civitas members, that cover a range of supplemental topics. 
  
We will not make ANY responses to questions publicly available or attribute responses to any specific organization. These data will only be presented in aggregate and will be published in a peer-reviewed journal (which we will be happy to send to you) and other publicly available publications and presentations. Please see below for more details on data access and data reporting.

**Data Access: Who Will Have Access to Individual, Identified Survey Responses**
The Civitas leadership team and the UCSF research team that are collecting the data will have access to fully identified survey responses.  In addition, the Office of the National Coordinator for Health IT (ONC) that is funding the survey will be given a dataset containing identifiable survey responses in the first five sections only. ONC may choose to share all or part of the dataset with ONC contractors only for the purpose of conducting contracted work and abiding by the same reporting/disclosure terms as described below. The sixth section will only be made available to Civitas and the UCSF research team.
  
**Data Reporting: What Data & Derivative Results Will be Reported in Journals, Data Briefs, or Public Documents**
No individual respondents or responses will ever be identified or reported.  All data will be reported at an aggregate level (e.g., across all survey responses).  For example, we may report that 10% of HIOs in the US have payers as participants.  A subset of data may be reported at the regional level (i.e., aggregated by state or healthcare market/HRR). Civitas, UCSF, ONC, and any ONC contractors receiving the data will abide by these terms.

**Screening Questions**

We would first like to ask you about the type of organization for which you are responding:

1. As of March 1, 2022, was your organization: (select one)

Supporting* “live” electronic health information exchange across your network

Building (or planning for) the infrastructure or services to support*, or pilot testing, electronic health information exchange across your network (End of survey)

No longer pursuing or supporting* electronic health information exchange (End of survey)

Never pursued or supported* electronic health information exchange (End of survey)

2. Does electronic health information exchange take place between independent entities**?

Yes

No (End of survey)

* Supporting is defined as offering a technical infrastructure that enables electronic health information exchange to take place.

**Independent entities are defined as institutions with different tax identification numbers; HIE between independent entities requires that ***at least one*** entity is independent of the other(s).

**Organizational Demographics**

1. Which of the following general categories apply to your organization: (Select all that apply)

Multi-state HIE

Single, statewide HIE

Community or local HIE

Governmental, state-designated HIE

Non-governmental, state-designated HIE

Enterprise HIE (i.e. primarily facilitate exchange between strategically aligned organizations)

Health Information Service Provider (HISP)

Other (please list):

1. What is your legal organizational structure?

State Government/Agency

Private Non-Profit 501c3

Private For-Profit

Other (please specify):

1. Since January 1, 2020, have you merged or are you planning to merge with another HIE?

No, not planning to do so

Currently considering

Yes, plan to merge. If public, with whom:

Yes, recently merged. If public, with whom:

1. *Which state(s) do you consider the primary ones in which you currently have, or are recruiting new, participants in your HIE? This should ***not*** include state(s) that you connect to via regional/national networks, such as Patient Centered Data Home or eHealth Exchange, or state(s) in which you provide technology for other HIEs that are branded under a different name.

Alabama  Alaska  American Samoa  Arizona

Arkansas  California  Colorado  Connecticut

Delaware  Distr. of Columbia  Florida  Georgia

Guam  Hawaii  Idaho  Illinois

Indiana  Iowa  Kansas  Kentucky

Louisiana  Maine  Maryland  Massachusetts

Michigan  Minnesota  Mississippi  Missouri

Montana  Nebraska  Nevada  New Hampshire

New Jersey  New Mexico  New York  North Carolina

North Dakota  N. Mariana Islands  Ohio  Oklahoma

Oregon  Pennsylvania  Puerto Rico  Rhode Island

South Carolina  South Dakota  Tennessee  Texas

Utah  US Virgin Islands  Vermont  Virginia

Washington  West Virginia  Wisconsin  Wyoming

1. 5a. *For the state(s) selected in question 4, please select the specific hospital service area(s) ^†^ in which you currently have, or are recruiting new, participants in your HIE.
   ^†^ Hospital Service Areas are geographic areas defined by the Dartmouth Atlas.
   *[Populate list of HSAs for each State reported in prior question and have check all option for HSAs in a given state]*

A hospital service area look-up by zip code can be found at: www.dartmouthatlas.org/data/search_zip.php

If you describe your service area differently or have additional comments on geographic area covered, please comment:

5b. If you have participants in other states or connections to HIEs in other states, please list those states here:

1. For the state(s) selected in question 4, what is the state’s general approach to consent?
   [Populate with states from question 4, limiting to those only reporting 1-5 states.]

Opt-in

Opt-out

Other (please specify):

Please indicate which of the following options applies to your HIE model:

Federated

Centralized

Both (Hybrid)

Other (please specify)

1. Which of the following services do you currently offer that are used by participants in your HIE? (Select all that apply)

| **GENERAL SERVICES** |  |
| --- | --- |
| Provider Directory |  |
| Patient Consent Management |  |
| Community Medical Record: Aggregation of information from across the community served by the HIE, only including health information (e.g., diagnoses, procedures, medications) |  |
| Community Health Record: Aggregation of information from across the community served by the HIE, including health and non-health information (e.g., transportation, education, and/or housing data) |  |
| Record Locator Service |  |
| Messaging using the Direct Protocol |  |
| Transform other document types or repositories into CCDAs (e.g., MDS, OASIS, Community Health Record) |  |
| Data normalization |  |
| Alerting/event notification (e.g., Admit-Discharge-Transfer) |  |
| Results delivery (i.e., uni-directional push) |  |
| Connection to prescription drug monitoring program (PDMP) |  |
| Prescription fill status and/or medication fill history |  |
| Provide data to third party disease registries (e.g., Wellcentive, Crimson) |  |
| Advanced care planning (i.e., POLST/MOLST) |  |
| Sell de-identified data to third parties |  |
| Patient access to immunization history |  |
| Integrating claims data |  |
| Other (please list): |  |

| **Services related to VALUE-BASED PAYMENT MODELS** |  |
| --- | --- |
| Activities related to quality measurement (e.g., generating, validating, reporting, etc.) |  |
| Closed-loop referrals tracking |  |
| Identification of gaps in care |  |
| Care coordination platform |  |
| Registry services, including operating as a clinical data registry or qualified clinical data registry (QCDR)^[[1]](#footnote-1)^ |  |
| Providing data to allow analysis by networks/providers |  |
| Analytics (e.g., risk stratification) |  |
| Other (please list): |  |

1. Do **entities participating in your HIE** **cover** 100% of your operating expenses?

Yes

No

1. Have you received HITECH 90/10 funds for implementation either directly as state designated entity, or indirectly through another entity?

Yes

No

Don’t know

1. Has your state Medicaid organization ever provided funding to support your HIE?

Yes – initial, one-time funding only

Yes – ongoing funding only

Yes – both initial and ongoing funding

In the process of obtaining approval for funding

No

Other: Please explain:

1. Does your HIE formally partner with your state Medicaid organization to provide data for quality reporting?

Yes, our HIE provides data for state quality reporting only

Yes, our HIE provides data for federal quality reporting only

Yes, our HIE provides data for state and federal quality reporting

We are in the process of working with state Medicaid to provide data for quality reporting

No

Other: Please explain:

1. If you have a **Master Patient Index (MPI)**, please ESTIMATE:

Total number of unique (resolved) individuals in your MPI:        Do not know

Total number of unique individuals in your MPI **with more than only demographic data**:        Do not know

1. Within the past year, please estimate **the number of acute care hospitals** (individual facilities both within health systems and independent, including VA, public, and private) that are directly connected (not via another network) to your HIE:

|  | HOSPITALS |
| --- | --- |
| Provide data | Do not know |
| Receive or view data | Do not know |

**Public Health**

***HIE Support for Public Health Reporting***

Screening: Is your HIE connected to any state of local public health entities? (Connected means that the public health entity provides data to your HIE, receives data from your HIE, and/or pays to participate in your HIE)

Yes

No (skip to next section)

Please list up to 5 state or local public health entities that are connected to your HIE:

|  |
| --- |
|  |
|  |
|  |
|  |

1a. For the entity(ies) listed, which type is each public health entity?

| **Answer Options**  *populate from those listed above* | **State Public Health Agency** | **Local or County Public Health Agency** | **Other** |
| --- | --- | --- | --- |
|  |  |  |  |
|  |  |  |  |
|  |  |  |  |
|  |  |  |  |
|  |  |  |  |

1b. For the entity(ies) listed, please report whether each public health entity**:** (Select all that apply)

| **Answer Options**  *populate from those listed above* | **… provides data to your HIE** | **Your HIE reports data to …** | **… pays to participate in your HIE** | **None of these options** |
| --- | --- | --- | --- | --- |
|  |  |  |  |  |
|  |  |  |  |  |
|  |  |  |  |  |
|  |  |  |  |  |
|  |  |  |  |  |

1c. Please report whether **the Centers for Disease Control and Prevention (CDC)**: (Select all that apply)

Provides data to your HIE

Receives reported data from your HIE

None of the above

If any option in column 2 of question 1b is selected:

**For questions 2-6 please answer for the PRIMARY public health agency (PHA) to which you are currently reporting data or are establishing ability to report data:**

1. Please indicate which you consider to be the primary public health agency to which you are currently or planning to establish reporting:

2a. Which of the following reporting services to your **primary public health agency (PHA)** do you offer to **your participating healthcare providers**?

|  | **In production** | **In testing** | **In planning** | **Available, but PHA not able/willing** | **Not available** | **Don’t know** |
| --- | --- | --- | --- | --- | --- | --- |
| Syndromic surveillance reporting |  |  |  |  |  |  |
| Immunization registry reporting |  |  |  |  |  |  |
| Electronic case reporting |  |  |  |  |  |  |
| Electronic reportable laboratory result reporting |  |  |  |  |  |  |
| Public health registry reporting (administered by or for public health agencies for public health purposes) |  |  |  |  |  |  |
| Clinical data and/or specialized registry reporting (administered by or for non-public health agency entities for clinical care and monitoring health care quality and resource use) |  |  |  |  |  |  |
| Other COVID-19 related reporting (e.g., registry) |  |  |  |  |  |  |
| Vital Record System reporting |  |  |  |  |  |  |

1. For each type of reporting to the primary PHA that is in production, are any of the following provider types currently using these services (i.e., at least one organization providing data for reporting)? (Select all that apply)

|  | **Hospitals** | **Office-based physicians** | **LTPAC settings** | **Urgent Care** | **Other** |
| --- | --- | --- | --- | --- | --- |
| Syndromic surveillance reporting |  |  |  |  |  |
| Immunization registry reporting |  |  |  |  |  |
| Electronic case reporting |  |  |  |  |  |
| Electronic reportable laboratory result reporting |  |  |  |  |  |
| Public health registry reporting |  |  |  |  |  |
| Clinical data registry reporting and/or specialized registry reporting |  |  |  |  |  |
| Other COVID-19 related reporting (e.g., registry) |  |  |  |  |  |
| Vital Record System reporting |  |  |  |  |  |

3a. For each type of reporting for ‘Other’ provider types, please indicate which provider types below.

|  | **Other Provider Types Reporting through your HIE** |
| --- | --- |
| Syndromic surveillance reporting |  |
| Immunization registry reporting |  |
| Electronic case reporting |  |
| Electronic reportable laboratory result reporting |  |
| Public health registry reporting |  |
| Clinical data registry reporting and/or specialized registry reporting |  |
| Other COVID-19 related reporting (e.g., registry) |  |
| Vital Record System reporting |  |

1. Do you receive any of the following funding source(s) to specifically support public health reporting? (Select all that apply)

Fees paid by participants

Fees paid by State health department

State Medicaid funding

STAR HIE program

CARES Act funding

Other Federal funding

Other State funding, including from State health department

Other. Please list:

Do not receive any funding to specifically support public health reporting

1. To what extent have you experienced the following barriers to public health reporting? This includes both reporting to and receiving from primary PHA.

|  | **To a Great Extent** | **Somewhat** | **Very Little** | **Not at All** | **N/A** |
| --- | --- | --- | --- | --- | --- |
| Patient consent model hinders data exchange with PHA |  |  |  |  |  |
| State statutes/regulations limit PHA participation with HIE |  |  |  |  |  |
| Need for data use agreements for public health data |  |  |  |  |  |
| Limited funding from PHA |  |  |  |  |  |
| Limited funding from your HIE participants |  |  |  |  |  |
| PHA lacks staffing |  |  |  |  |  |
| PHA lacks technical capability to receive messages from your HIE |  |  |  |  |  |
| PHA lacks technical capability to process messages from your HIE |  |  |  |  |  |
| Other technical limitations on part of PHA |  |  |  |  |  |
| PHA has other priorities |  |  |  |  |  |
| Low return on investment to your HIE |  |  |  |  |  |
| Other (please list): |  |  |  |  |  |

1. Since February 2020, have you expanded the number of provider organizations that engage in public health reporting through your HIE?

Yes

No

Don’t know

6a. If yes, Which provider types expanded public health reporting through your HIE? (Select all that apply)

| Hospitals and Health Systems |  |
| --- | --- |
| Ambulatory Clinics/Physician Practices |  |
| Long-term Care Facilities |  |
| Correctional Facilities |  |
| Labs (commercial, public health) |  |
| Behavioral Health Providers |  |
| Other (please list): |  |

If any option in column 1 of question 1b is selected:

7a. Which of the following types of data do you **receive** from public health entities with which you have established connectivity? (Select all that apply)

Syndromic surveillance

Immunization

Electronic case reports

Electronic reportable laboratory results

Data from public health registry (administered by or for public health agencies for public health purposes)

Data from clinical data and/or specialized registry (administered by or for non-public health agency entities for clinical care and monitoring health care quality and resource use)

Data related to COVID-19

Vital records

Other. Please list:

Don’t know

7b. What is the purpose of receiving public health data? (Select all that apply)

To identify opportunities to enrich public health data with HIE data

To make public health data available to your participants

Other. Please list:

***HIE Support for Public Health Exchange Related to the Pandemic***

What are your current capabilities to electronically receive hospital data on **bed capacity and resource utilization**? Electronic receipt includes standards-based approaches (e.g., SANER, HL7 feed) and does **not** include spreadsheet submission and/or manual data entry.

Actively electronically receiving production data

In the process of testing and validating electronic receipt of data (Skip to 9)

In planning phase to support this reporting (Skip to 9)

Not planning to support this reporting (Skip to 9)

Don’t know (Skip to 9)

8a. If actively electronically receiving production data, to what entities are you submitting this data? (Select all that apply)

City or local public health department(s)
 State public health department(s)
 Federal entities (such as, the CDC or HHS)
 Other. Please list:      
 Don’t know

8b. How do hospitals transmit hospital capacity and resource utilization data to your HIE? (Select all that apply)

ADT messages

HL7 v2 messages

SANER FHIR Server <https://build.fhir.org/ig/HL7/fhir-saner/introduction.html>

Other. Please list:

Don’t know

8c. What terminology standards are used by hospitals to report hospital capacity and resource utilization data? (Select all that apply)

NIEM

LOINC

Other. Please list:

Non-standardized codes

Don’t know

1. Does your HIE currently provide data to PHA(s) to fill gaps in their COVID-19-related data (e.g., missing demographic information)?

Yes

No but could do so

No and could not do so

Don’t know

9a. If yes or could do so: Please indicate what types of data are or could be provided to fill gaps. (Select all that apply)

| Data Type | Currently provided | Not currently provided but could be |
| --- | --- | --- |
| Race/ethnicity |  |  |
| Other demographics |  |  |
| Up-to-date contact information (for contact tracing) |  |  |
| Hospitalization information |  |  |
| Health information such as chronic health conditions |  |  |
| Immunization data |  |  |
| Commercial lab results |  |  |
| Hospital lab results |  |  |
| Other: |  |  |

9b. If yes: How often do PHA(s) electronically receive or query these types of data from your HIE?

Often

Sometimes

Rarely

Never

Don’t know

9c. If yes: How are PHA(s) accessing these data? (Select all that apply)

Single patient lookup through a Portal

Batch query and response

API

Aggregate data and/or statistics (e.g., dashboard)

SFTP/Amazon S3 file transfer

Other. Please list:

Not applicable

1. What other services does your HIE provide to PHA(s) to support COVID-19 response: (Select all that apply)

Analytic and Data Quality Support (beyond those reported above)

Dashboarding and Data Visualization Assistance

Process Automation

Bidirectional Data Sharing/Receiving Data from PHAs

Use of HIE MPIs to Support Public Health Deduplication or Other Services

Outbreak Monitoring and Alerting

Public Health Policy Impact Monitoring

Other. Please list:

None

1. Do PHA(s) contribute COVID-19 immunization registry data or make COVID-19 immunization registry data available for query through your HIE?

Yes

No

Don’t know

1. Other than PHAs, who are the users of your HIE’s COVID-19 data? (Select all that apply)

Healthcare Providers: Administrators  Emergency Preparedness/Response

Healthcare Providers: Frontline Clinicians  School Nurses

Payers  Contact Tracers

Medicaid  CDC

Other. Please list:

None

If ‘Healthcare Providers: Frontline Clinicians’ is checked: What COVID-19 data can frontline clinicians access through your HIE: (Select all that apply)

COVID-19 test results/case status

COVID-19 antibodies

Other respiratory illness history

Vaccination Status

Hospital Status/Capacity Information

Healthcare utilization (inpatient, outpatient, EHR visits, etc.)

Demographics (age, race, ethnicity, etc.)

Other. Please list:

Don’t know

If ‘Healthcare Providers: Frontline Clinicians’ is checked: How can frontline clinicians access COVID-19 data through you HIE: (Select all that apply)

Individual patient look-up via portal or query

De-identified reports

Bulk query for identified data on populations

Dashboards and interactive reporting

Public or private briefings on community/statewide COVID-19 status

Secure email notifications

Other. Please list:

Don’t know

***Lab Participation in COVID-19 Relevant HIE***

1. Please report whether **each type of stakeholder is involved** in your HIE in the following ways:

| **Answer Options** | **Provide COVID-19 Test Results** | **Provide Data Other Than COVID-19 Test Results** | **View or Receive Data** | **Not involved** |
| --- | --- | --- | --- | --- |
| Hospital-based labs |  |  |  |  |
| Independent labs (including commercial) |  |  |  |  |
| Physician office-based labs |  |  |  |  |
| Mobile labs (e.g., Point of Care Labs for COVID-19) |  |  |  |  |
| Public health labs |  |  |  |  |
| Other: |  |  |  |  |

1. If ‘Provide COVID-19 Test Results’ is checked, for the relevant rows: How timely are COVID-19 test results that you typically receive?

|  | Real Time; Near Real Time | Within 24 hours | Greater than 24 hours but less than 48 hours | Greater than 48 hours | Don’t Know | Not applicable |
| --- | --- | --- | --- | --- | --- | --- |
| Hospital-based labs |  |  |  |  |  |  |
| Independent labs (including commercial) |  |  |  |  |  |  |
| Physician office-based labs |  |  |  |  |  |  |
| Mobile labs (e.g., Point of Care Labs for COVID-19) |  |  |  |  |  |  |
| Public health labs |  |  |  |  |  |  |
| Other: |  |  |  |  |  |  |

1. In general, have laboratories sought to limit or refused to provide access, exchange, or use of electronic health information (e.g., laboratory results)?

Yes

No (skip to 21)

Have not made request (skip to 21)

1. What types of laboratories have sought to limit or refused to provide access, exchange, or use of electronic health information? (Select all that apply)

Hospital-based labs

Independent labs (including commercial)

Physician office-based labs

Mobile labs (e.g., Point of Care Labs for COVID-19)

Public health labs

Other. Please list:

1. Which of the following reasons have laboratories used as the basis for limiting or refusing to provide electronic health information to your HIE? (Select all that apply)

Role of CLIA or other federal regulations in restricting them from sending additional data

Fees associated with HIE participation

Labs don’t derive value as a data contributor only

Concerns with HIE’s ability to do patient matching

Concerns with producing duplicate data

Exchanging data with HIEs is not considered related to treatment, payment, or operations and thus would require patient consent

Labs reporting obligation ends with returning result to ordering provider

Public health agencies (including emergency rules) do not mandate reporting to HIE

Labs need consent from each individual provider, resulting in your HIE having to execute multiple disclosure forms (e.g., for each participating health care provider)

Technological reasons/use of specific standards (convenient reason or wide spectrum of what labs are able to do)

Other. Please list:

1. To what degree have you been able to overcome these difficulties to access data from laboratories?

Not at all

To a small extent

Somewhat

To a great extent

Fully

1. Does your HIE map from non-standard laboratory test/result codes to LOINC® codes?

Yes

No (Skip to next section)

Don’t know (Skip to next section)

21a. Within the past year, based upon the volume of test results received (qualitative and quantitative), to what extent did your HIE have to map those results from non-standard codes to LOINC codes?

All or most

Some

Few

None

Don’t know

21b. Have you experienced any of the following issues related to mapping to LOINC? (Select all that apply)

We do not have sufficient expertise to map to LOINC within our organization

We find LOINC and LOINC tools too difficult to use

We do not have the resources (personnel/time) to map to and/or maintain mappings to LOINC

Other issue. Please specify:

No, we have not experienced any issues mapping to LOINC

Don’t know

**Implementation and Use of Standards**

1. To what extent does your HIE electronically **receive** data from your participants using the following methods listed below? (Select one option across a row)

*Please consider the methods used by participant to provide the data to your HIE. Do not include conversions you may do after receipt. With regards to conformance to standards, if the receipt of the data is in partial conformance, please consider that as conformant.*

|  | **Routinely/**  **from most participants** | **Sometimes/**  **From some participants** | **Rarely/**  **From few participants** | **Never** | **Don’t know** |
| --- | --- | --- | --- | --- | --- |
| Care summaries in a structured format (e.g., CDA, CCR, C32) |  |  |  |  |  |
| HL7 v2 messages (any type) |  |  |  |  |  |
| ADT messages (for applicable participants) |  |  |  |  |  |
| HL7 Fast Healthcare Interoperability Resources (FHIR) messages (DSTU2) |  |  |  |  |  |
| HL7 FHIR Release 3 (STU) messages |  |  |  |  |  |
| FHIR v.4.0 messages |  |  |  |  |  |

1a. If care summaries in a structured format “routinely” or “sometimes” is checked above, then ask: Do you parse C-CDAs (i.e., extract and make available discrete data elements):

Yes

No

Don’t know

1. To what extent is the information that you **receive from your participants** consistent with different versions of the United States Core Data for Interoperability (USCDI)? *USCDI is a standardized set of health data classes and constituent data elements for nationwide, interoperable health information exchange.*

|  | **Routinely/**  **from most participants** | **Sometimes/**  **From some participants** | **Rarely/**  **From few participants** | **Never** | **Don’t know** |
| --- | --- | --- | --- | --- | --- |
| USCDI v1 <https://www.healthit.gov/isa/united-states-core-data-interoperability-uscdi> |  |  |  |  |  |
| USCDI v2  <https://www.healthit.gov/isa/united-states-core-data-interoperability-uscdi#uscdi-v2> |  |  |  |  |  |

1. To what extent does your HIE electronically **send or make available** data to your participants using the following methods?

|  | **Routinely/**  **To most participants** | **Sometimes/**  **To some participants** | **Rarely/**  **To few participants** | **Never** | **Don’t know** |
| --- | --- | --- | --- | --- | --- |
| Care summaries in a structured format (e.g., CDA, CCR, C32) |  |  |  |  |  |
| HL7 v2 messages (any type) |  |  |  |  |  |
| HL7 Fast Healthcare Interoperability Resources (FHIR) messages DSTU2 |  |  |  |  |  |
| HL7 FHIR Release 3 (STU) |  |  |  |  |  |
| FHIR v.4.0 |  |  |  |  |  |

1. Which types of **clinical and other health-related information** are made available by your HIE (as part of a clinical document or as a structured data element)? (Select all that apply)

|  | **Included in your HIE** |
| --- | --- |
| Data Provenance |  |
| **Clinical Information** | |
| Problems |  |
| Prescribed Medications |  |
| Filled Medications |  |
| Medication Allergies |  |
| Non-Medication Allergies & Intolerances |  |
| Functional Status |  |
| Cognitive Status |  |
| Vital Signs |  |
| Pregnancy Status |  |
| Immunizations |  |
| Family Health History |  |
| Health Concerns |  |
| Clinical Notes |  |
| **Imaging/Pathology** | |
| Diagnostic Imaging Order |  |
| Radiology Report (narrative) |  |
| Pathology Report (narrative) |  |
| **Laboratory-Related Information** |  |
| Laboratory Test(s) |  |
| Laboratory Value(s)/Result(s) |  |
| Laboratory report (narrative) |  |
| **Team-Based Care** | |
| Care Plan Field(s), including Goals and Instructions |  |
| Care Team Member(s)  (Provider ID, Provider Name) |  |
| Assessment and Plan of Treatment |  |
| **Encounter-Related Information** | |
| Procedures |  |
| Admission and Discharge Dates and Locations |  |
| Encounters (Encounter type, diagnosis, time) |  |
| Discharge Disposition |  |
| Referrals |  |
| Discharge Instructions |  |
| Reason for Hospitalization |  |
| **Health Equity** | |
| Home Address |  |
| Race/Ethnicity |  |
| Preferred Language |  |
| Health-related Social Needs (e.g., housing, food insecurity) |  |
| Substance Use Disorder (as defined in 42 CFR Part 2) |  |
| Gender Identity |  |
| Sexual Orientation |  |
| **Other** | |
| Other (please list): |  |

To what extent does your HIE electronically **send or make available to participants**:

|  | **Routinely/**  **To most participants** | **Sometimes/**  **To some participants** | **Rarely/**  **To few participants** | **Never** | **Don’t know** |
| --- | --- | --- | --- | --- | --- |
| Care summaries in a structured format (e.g., CDA, CCR, C32) |  |  |  |  |  |
| Data in a format consistent with USCDI v1 |  |  |  |  |  |
| Data in a format consistent with USCDI v2 |  |  |  |  |  |

Does your HIE **make data available for participants to query?** Note: query refers to a query-and-response exchange, e.g. a request from one participant through an interface that results in a response delivered into an EHR.

Yes

No

Don’t Know

**Network-to-Network Connectivity and TEFCA**

1. Does your HIE: (Select all that apply)

|  |  |
| --- | --- |
| Sell/provide your infrastructure to other HIEs |  |
| Buy/use infrastructure from another HIE |  |
| Connect to other HIEs in SAME state |  |
| Connect to other HIEs in DIFFERENT state(s) |  |
| None of the above |  |

1. Is your HIE currently using the following national networks to exchange data?

|  | **Live Data Exchange (send or receive)** | **Implementing** | **Not Using** | **Other (please specify):** |
| --- | --- | --- | --- | --- |
| **General Purpose Networks:** |  |  |  |  |
| CommonWell |  |  |  |  |
| DirectTrust |  |  |  |  |
| Strategic Health Information Exchange Collaborative (Civitas)/Patient Centered Data Home |  |  |  |  |
| e-Health Exchange |  |  |  |  |
| Carequality |  |  |  |  |
| **Specific Purpose Networks:** |  |  |  |  |
| Surescripts |  |  |  |  |
| Patient Ping |  |  |  |  |
| Audacious Inquiry: Pulse/ENS |  |  |  |  |
| Collective Medical Technologies: EDIE |  |  |  |  |
| Social Service Referral Platform(s) (e.g., Aunt Bertha, Unite Us) |  |  |  |  |
| Other (please list): |  |  |  |  |

2a. If not using any general-purpose networks in prior question: Please select reason(s) for not using any of the general purpose networks: (Select all that apply)

Do not see the value in what they provide (i.e., services not useful or data limited)

Perceive them as competitors

Participation costs too high

Not a priority

Other. Please list:

1. Is your HIE planning to participate in the Trusted Exchange Framework and Common Agreement? Please find definitions of the roles here: <https://www.healthit.gov/topic/interoperability/trusted-exchange-framework-and-common-agreement-tefca>

Yes, as a QHIN

Yes, as a Participant or as a Sub-participant

No

Don’t know

3a. If no: Why are you not planning on participating in TEFCA? (Select all that apply)

Don’t have enough information

Don’t have time/resources to prepare

Concerns about the terms of the Common Agreement (please briefly describe):       
 Concerns over privacy and/or security of the network

Concerns about the burden associated with participation (e.g., financial, reporting) (please briefly describe):

Do not perceive sufficient value in participating (please briefly describe why):       .

Other (please list):

3b. If don’t know: Why are you unsure about participating in TEFCA? (Select all that apply)

Don’t have enough information

Don’t have time/resources to prepare

Concerns about the terms of the Common Agreement (please briefly describe):       
 Concerns over privacy and/or security of the network

Concerns about the burden associated with participation (e.g., financial, reporting) (please briefly describe):

Do not perceive sufficient value in participating (please briefly describe why):

Have not yet developed a strategic plan to participate

Other (please list):

3c. If yes: As exchange based on the Trusted Exchange Framework and Common Agreement becomes operational, is your HIE planning to change its operations in any of the following ways:

|  | **Yes** | **No** | **Don’t know** | **Not Applicable** |
| --- | --- | --- | --- | --- |
| Changing Types of services offered |  |  |  |  |
| Selling/providing your services to other HIEs |  |  |  |  |
| Buying/using services from another HIE |  |  |  |  |
| Changing technical infrastructure |  |  |  |  |
| Changing legal agreements and/or policies |  |  |  |  |
| Changing other infrastructure (e.g., creating new training, supporting or making process redesigns (e.g., new workflows)) |  |  |  |  |
| Partnering with HIEs in SAME region/state |  |  |  |  |
| Partnering with HIEs in DIFFERENT regions/states |  |  |  |  |
| Partnering with an entity that is not an HIE (e.g., Health IT Developer) |  |  |  |  |
| Other (please list): |  |  |  |  |

4a. For which of the following exchange purposes (which are included in TEFCA), are your participants currently able to make a REQUEST for information?

|  | **Yes** | **No** | **Don’t Know** |
| --- | --- | --- | --- |
| Treatment (as defined by HIPAA) |  |  |  |
| Payment (as defined by HIPAA) |  |  |  |
| Health Care Operations (as defined by HIPAA) |  |  |  |
| Individual Access |  |  |  |
| Public Health |  |  |  |
| Government Benefits Determination (as defined by TEFCA) |  |  |  |

4b. For which of the following exchange purposes (which are included in TEFCA), are your participants currently able to RESPOND WITH ADEQUATE DATA to a Request for information?

|  | **Yes** | **No** | **Don’t Know** |
| --- | --- | --- | --- |
| Treatment (as defined by HIPAA) |  |  |  |
| Payment (as defined by HIPAA) |  |  |  |
| Health Care Operations (as defined by HIPAA) |  |  |  |
| Individual Access Services |  |  |  |
| Public Health |  |  |  |
| Government Benefits Determination (as defined by TEFCA) |  |  |  |

**Information Blocking**

Information blocking practices have been defined in rules that went into effect on April 5, 2021. The following set of questions ask about practices that may constitute information blocking based on your understanding of the rules. Please respond based on your experience since the rules went into effect (April 5, 2021).

1. To what extent are you familiar with the information blocking rules, applicable actors, exceptions, and enforcement timeline?

Very Familiar

Moderately Familiar

Somewhat Familiar

Not Familiar

1. How often have you encountered **each of the following form(s)** of information blocking by **EHR vendors** (and other Developer(s) of Certified Health IT)?

|  | **Rarely/Never** | **Sometimes** | **Often/ Routinely** | **Don’t Know** |
| --- | --- | --- | --- | --- |
| **PRICE**  Examples:  using high fees to avoid granting third-parties access to data stored in the developer’s EHR system  charging unreasonable fees to export data at a provider’s request (such as when switching developers) |  |  |  |  |
| **CONTRACT LANGUAGE**  Examples:  using contract terms, warranty terms, or intellectual property rights to discourage exchange or connectivity with third-party  changing material contract terms related to health information exchange after customer has licensed and installed the vendor’s technology |  |  |  |  |
| **ARTIFICIAL TECHNICAL, PROCESS, OR RESOURCE BARRIERS**  Examples:  using artificial technical barriers to avoid granting third-parties access to data stored in the vendor’s EHR system  using artificial reasons to limit the types of information that can be sent/shared or received |  |  |  |  |
| **REFUSAL**  Examples:  refusing to exchange information or establish connectivity with certain vendors or HIOs  refusing to export data at a provider’s request (such as when switching vendors) |  |  |  |  |
| **OTHER** (please list): |  |  |  |  |

1. What proportion of **EHR Vendors** have you encountered engaging in information blocking?

All/Most

Some

Few

None (skip to 6)

Don’t know or N/A (Don’t interact with developers) (skip to 6)

3a. Among **EHR Vendors** that engage in information blocking, how often do they do it?

Routinely

Sometimes

Rarely

Don’t know

1. When you have experienced practices that you believed constituted information blocking by **EHR vendors** in the past year, how often did you report the information blocking to ONC/HHS?

Always

Most of the time

Sometimes

Rarely

Never

1. To what extent does information blocking by **EHR vendors** make it more difficult for you to provide HIE services to your participants?

Greatly

Moderately

Minimally/Not at all

Don’t know

1. In what form(s) have you experienced information blocking by **hospitals and health systems**?

|  | **Rarely/Never** | **Sometimes** | **Often/ Routinely** | **Don’t Know** |
| --- | --- | --- | --- | --- |
| **ARTIFICIAL TECHNICAL, PROCESS, OR RESOURCE BARRIERS**  Examples:  requiring a written authorization when neither state nor federal law requires it  requiring a patient to repeatedly opt in to exchange for TPO |  |  |  |  |
| **REFUSAL**  Examples:  refusing to exchange information with competing providers, hospitals, or health systems  refusing to share data with other stakeholders, such as payers or independent labs |  |  |  |  |
| **CLOSED NETWORK EXCHANGE**  Examples:  promoting alternative, proprietary approaches to HIE  exchanging only within referral network or with preferred referral partners |  |  |  |  |
| **OTHER** (please list): |  |  |  |  |

1. What proportion of **hospitals and health systems** have you encountered engaging in information blocking?

All/Most

Some

Few

None (skip to 9)

Don’t know or N/A (Don’t interact with developers) (skip to 9)

7a. Among **hospitals and health systems** that engage in information blocking, how often do they do it?

Routinely

Sometimes

Rarely

Don’t know

1. To what extent does information blocking by **hospitals and health systems** lead to missing patient health information?

Greatly

Moderately

Minimally/Not at all

Don’t know

1. Among other types of stakeholders, to what extent have you observed information blocking behaviors?

|  | **Rarely/Never** | **Sometimes** | **Often/ Routinely** | **Don’t Know** |
| --- | --- | --- | --- | --- |
| **Commercial Payers** |  |  |  |  |
| **Commercial Laboratories** |  |  |  |  |
| **Commercial Pharmacies** |  |  |  |  |
| **National Networks (e.g. CommonWell, eHealth Exchange)** |  |  |  |  |
| **State, regional, and/or local health information exchange** |  |  |  |  |
| **Other** (please list): |  |  |  |  |

1. Across all types of stakeholders, to what extent has information blocking decreased since the final regulations went into effect in April 2021?

Greatly

Moderately

Minimally/Not at all

Don’t know or N/A

**Additional Information**

1. Initiative or Organization Name:

2. We appreciate your participation. Would you like to receive a copy of our results that will enable you to compare your effort to others in the nation?

Yes

No

3. If you would like to receive a $50 amazon.com gift certificate, please complete the following fields:

Name:

Email:

1. A Qualified Clinical Data Registry (QCDR) is a Centers for Medicare & Medicaid Services (CMS) approved vendor that is in the business of improving health care quality. These organizations may include specialty societies, regional health collaboratives, large health systems or software vendors working in collaboration with one of these medical entities. [(CMS)](https://www.cms.gov/Medicare/Quality-Initiatives-Patient-Assessment-Instruments/MMS/Downloads/A-Brief-Overview-of-Qualified-Clinical-Data-Registries.pdf) [↑](#footnote-ref-1)
